# Integrated analysis of lncRNA-miRNA-mRNA ceRNA network in human aortic dissection

**DOI:** 10.1186/s12864-021-08012-3

**Published:** 2021-10-07

**Authors:** Hao Zhang, Ce Bian, Simei Tu, Fanxing Yin, Panpan Guo, Jian Zhang, Xiaotong Song, Qingyang Liu, Chen Chen, Yanshuo Han

**Affiliations:** 1 School of Life and Pharmaceutical Sciences, Dalian University of Technology, Dalian, China; 2 Department of Cardiovascular Surgery, The General Hospital of the PLA Rocket Force, Beijing Normal University, Beijing, China; 3 Department for Vascular Surgery, First Hospital of China Medical University, Shenyang, China; 4 School of Biomedical Sciences, The University of Queensland, Brisbane, Australia

**Keywords:** Aortic dissection, lncRNA, miRNA, ceRNA network

## Abstract

**Background:**

Many studies on long chain non-coding RNAs (lncRNAs) are published in recent years. But the roles of lncRNAs in aortic dissection (AD) are still unclear and should be further examined. The present work focused on determining the molecular mechanisms underlying lncRNAs regulation in aortic dissection on the basis of the lncRNA-miRNA-mRNA competing endogenous RNA (ceRNA) network.

**Methods:**

This study collected the lncRNAs (GSE52093), mRNAs (GSE52093) and miRNAs (GSE92427) expression data within human tissue samples with aortic dissection group and normal group based on Gene Expression Omnibus (GEO) database.

**Results:**

This study identified three differentially expressed lncRNAs (DELs), 19 differentially expressed miRNAs (DEmiRs) and 1046 differentially expressed mRNAs (DEGs) identified regarding aortic dissection. Furthermore, we constructed a lncRNA-miRNA-mRNA network through three lncRNAs (including two with up-regulation and one with down-regulation), five miRNAs (five with up-regulation), as well as 211 mRNAs (including 103 with up-regulation and 108 with down-regulation). Simultaneously, we conducted functional enrichment and pathway analyses on genes within the as-constructed ceRNA network. According to our PPI/ceRNA network and functional enrichment analysis results, four critical genes were found (E2F2, IGF1R, BDNF and PPP2R1B). In addition, E2F2 level was possibly modulated via lncRNA FAM87A-hsa-miR-31-5p/hsa-miR-7-5p or lncRNA C9orf106-hsa-miR-7-5p. The expression of IGF1R may be regulated by lncRNA FAM87A-hsa-miR-16-5p/hsa-miR-7-5p or lncRNA C9orf106-hsa-miR-7-5p.

**Conclusion:**

In conclusion, the ceRNA interaction axis we identified is a potentially critical target for treating AD. Our results shed more lights on the possible pathogenic mechanism in AD using a lncRNA-associated ceRNA network.

## Introduction

Aortic dissection (AD) is a severe aortic disorder involving destruction of aortic wall medial layer, which can induce separation between intima and adventitia, and track blood within the dissection plate in the medial layer, thereby inducing the formation of true and false lumens in the aortic wall. Although the research on aortic dissection has gradually increased and many innovations have been made in research programs, the treatment of thoracic aortic dissection is still extremely challenging [1, 2]. Aortic dissections are more common in the non-whites population of elderly men [3], and the incidence rate increases sharply over the age of 50. People between the ages of 50 and 70 are at the highest risk, and increasing age was the important variable associated with AD long-term mortality [4]. In the meanwhile, because it is often misdiagnosed when it appears, it is difficult to assess the exact incidence [5]. Although studies have demonstrated that the diagnostic techniques and treatments for thoracic AD cases are improving, the mortality and morbidity are high, so early diagnosis and treatment are very necessary [6].

The current research always draws their attention to the technical means and pathogenesis of aortic dissection. More and more recent studies have suggested that non-coding RNAs (ncRNAs), including long noncoding RNAs (lncRNAs) and microRNAs (miRNAs), exert decisive roles in the development of cardiovascular diseases (CVDs) [7, 8]. lncRNAs are the ncRNAs that are over 200 bp in length, which can be used as an important type of regulatory molecule in the human genome to perform its biological functions in various ways. Many studies have shown that IncRNA can also be used to be the competitive endogenous RNA (ceRNA) as the miRNA sponges and participate in regulating the expression of target genes [9, 10]. miRNAs shows negative regulation on protein-coding genes through combining with complementary sequences [11]. As a result, the lncRNA-miRNA-mRNA interaction has a vital effect on CVD development [12, 13].

Ren and colleagues revealed that miR-193b-3p and lncRNA H19 had certain effect on vascular smooth muscle cell (VSMC) migration and proliferation, which facilitated to generate new thoughts in AD management [14]. Recently, Zhang et al. carried out a study in order to prove lncRNA XIST’s effect on AD pathogenic mechanism and identify its corresponding pathway, with the findings showing that lncRNA XIST regulated smooth muscle cell proliferation and apoptosis through the sponge of miR-17 and the regulation of the subsequent downstream PTEN to affect the development of aortic dissection in mice [15]. Simultaneously, Zhao and colleagues suggested that lncRNA CDKN2B-AS1 regulated STAT3 level through suppressing miR-320d to regulate human VSMC apoptosis and proliferation [16]. To sum up, lncRNA-miRNA-mRNA regulatory network exerts a vital part in AD genesis and progression while the lncRNA targets, roles and underlying mechanisms within different tissues and cells of AD have not been reported. At the same time, there are few reports about the ceRNA regulation mechanism of lncRNA-miRNA related to AD and the interaction between ncRNAs.

In this study, lncRNAs together with the corresponding mechanisms of action within human tissue from AD cases were explored by means of bioinformatic analysis. Firstly, this study applied Gene Expression Omnibus (GEO) database for obtaining the AD-related lncRNA, mRNA and miRNA expression data. Thereafter, we discovered differentially expressed genes (DEGs), differentially expressed miRNAs (DEmiRs), and differentially expressed lncRNAs (DELs) using RStudio. Cytoscape 3.7.2 was utilized to construct a lncRNA-miRNA-mRNA network, followed by the construction of a PPI network.

The present study aims to further screen the key lncRNA-miRNA-mRNA ceRNA axis within AD with microarray data collected from public databases and bioinformatics methods. Besides, our results shed more lights on the AD molecular mechanism, thus providing a novel direction for targeted therapy of AD. To further examine the key gene regulatory axis, we established a lncRNA-miRNA-mRNA regulatory subnetwork.

## Materials and methods

### Microarray data and data processing

**Fig. 1:**
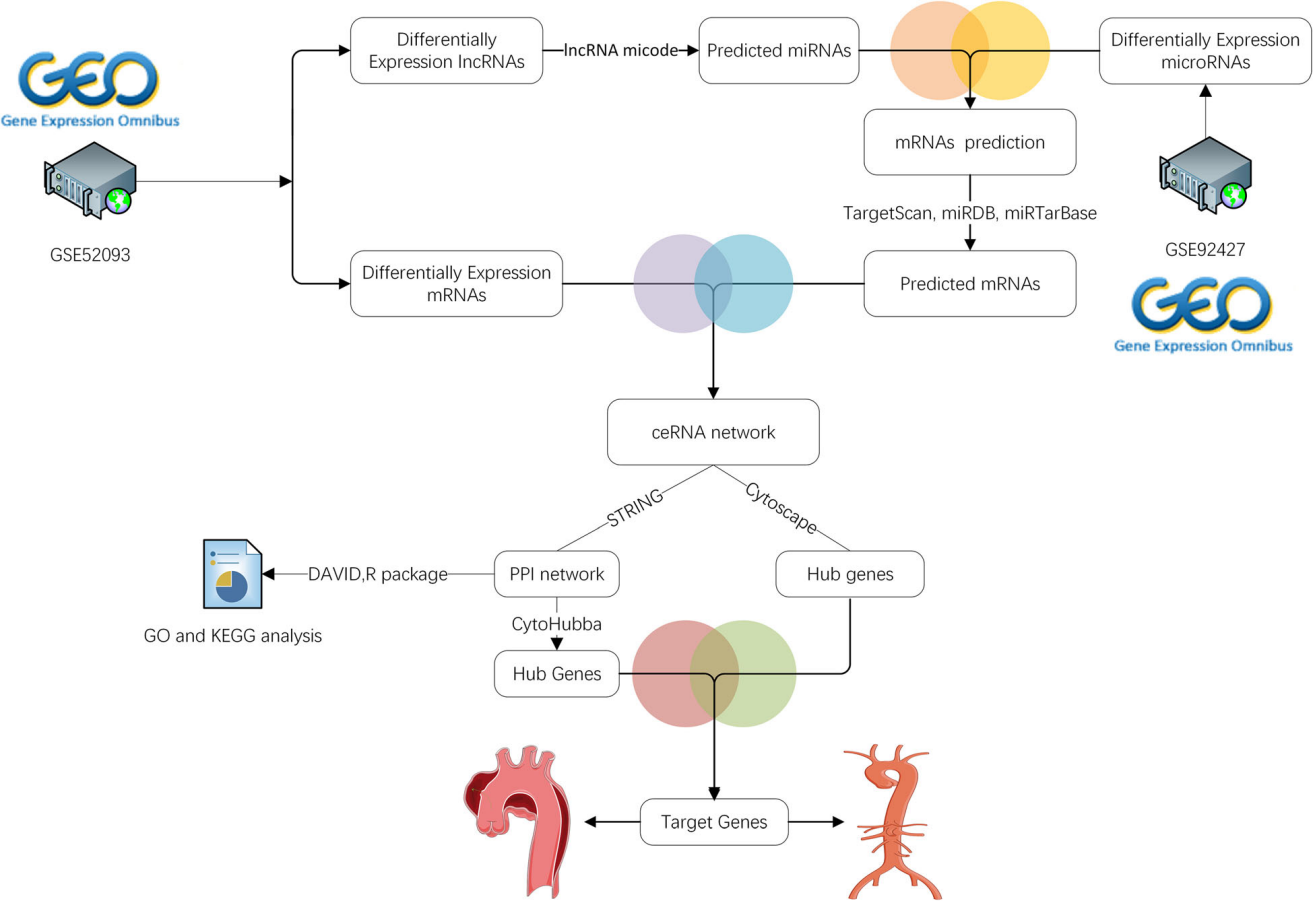
Flow diagram of data processing. The difference expression of two datasets (miRNA dataset, lncRNA dataset and mRNA dataset) was analyzed, and then the intersection was selected; Construction of CeRNA network, protein-protein interaction network and functional enrichment analysis, and finally determine the key genes

Two datasets were obtained by setting the screening criteria for the species type as “*Homo sapiens*”, from GEO database of National Center for Biotechnology Information (NCBI) (https://www.ncbi.nlm.nih.gov/geo/), with such keywords being searched as “Aortic dissection” (AD). Then select the study type as " Expression profiling by array", so that we get the series matrix files and platform file of non-coding RNA. as a result, a total of two datasets were included in this study, namely GSE52093 and GSE92427. As for lncRNA and mRNA expression data (GSE52093 dataset), relative to normal aortic tissue in control group, the experimental group collected the ascending aorta of patients with acute Stanford type A aortic dissection to identify differentially expressed genes. The microRNA microarray is used for expression profiling analysis from subjects of 24 plasma samples (including acute aortic dissection and healthy subjects). Each dataset contains aorta dissected group and aorta normal group. Simultaneously, we applied GEO database for downloading series matrix files and expressive data. The data flow mechanism diagram is displayed in (Figure 1).

### DEGs analysis

DEGs were analyzed by R package “Linear Models for Microarray Data (limma)” function for datasets and online analysis software SPSSAU. SPSSAU (https://spssau.com/index.html) is an online data analysis and visualization software. We inputted the expression matrix data of two datasets into SPSSAU and got the difference between aorta dissected group and aorta normal group of two datasets. In addition, we also obtained the median, extremum and outliers between each sample.

For each dataset, the sva R package was used to remove batch effects and the GeneSoring GX software package was adopted to conduct the quantile normalization for preprocessing, and the annotation information of lncRNA, miRNA and mRNA was downloaded by series matrix files of GSE52093 and GSE92427 datasets. The selection criteria for GSE52093 dataset including log|FoldChange|>1 and adjust*p*<0.1 were regarded as threshold values. The selection criteria for GSE92427 dataset containing log|FoldChange|>1 and adjust*p*<0.05 were regarded as threshold values. There existed statistical significance for the selection of this threshold, and those genes that were up- and down-regulated can also be selected for performing the subsequent analysis. This indicates statistical significance. R software package was also adopted as a heat map and volcano map drawing of DELs, DEmiRs and DEmRNAs. Subsequently, these DELs, DEmiRs and DEmRNAs between two databases were classified as the up-regulation or down-regulation group. These data would be used in the following ceRNA network construction and protein interaction network construction.

### Prediction of miRNA-mRNA and lncRNA-miRNA pairs

Interaction relationships between lncRNAs and miRNAs were predicted with R package and miRcode database. By using the R language program to output all the predicted results of DElncRNAs, and intersecting the output results with DEmiRs, the interaction pair of lncRNAs and miRNAs can be obtained. Cytoscape (version 3.7.2) was utilized for visualizing forecast results.

Similarly, we use the R package to predict the miRNAs in the lncRNA-miRNA interaction pair, and intersect the prediction result with the DEmRNAs. As a result, the miRNA-mRNA interaction pair can be obtained. The difference is that we use three databases when predicting mRNAs, and we extract the results with 2 occurrences in the three databases as our prediction result.

Interactions between lncRNAs and miRNAs were analyzed on the basis of lncRNA target prediction databases shown below:

miRcode (http://www.mircode.org/).

Interactions between miRNAs and mRNAs were analyzed in line with 3 miRNA target prediction databases:

- miRTarBase (https://maayanlab.cloud/Harmonizome/resource/MiRTarBase),
- TargetScan (http://www.targetscan.org/vert_72/),
- miRDB (http://mirdb.org/).
- Perl (version 5.32.0) was adopted to analyze data.

### Establishment of the ceRNA network of lncRNA-miRNA-mRNA

This study established a ceRNA network with related DELs by using commonly interactive miRNAs with DELs and DEmRNAs. Cytoscape (version 3.7.2) was later utilized to visualize the ceRNA network of lncRNA/miRNA/mRNA.

### PPI network analysis and key gene identification

The plug-in CytoHubba in the Cytoscape software is a visualization software that obtains the dense relationship through the degree, closeness centrality and betweenness centrality algorithms. Those hub genes in ceRNA network were identified by CytoHubba.

The present study adopted the Search Tool for the Retrieval of interacting Genes/Proteins (STRING; version 11.0) for retrieving protein interactions between DEGs identified in GSE52093. After the points without interaction were hidden, the data were imported into Cytoscape (version 3.7.2) for visualizing the protein–protein interaction (PPI) network. CytoHubba was used to identifiy the hub genes of PPI network.

The key genes were determined by taking the intersection of hub genes in the ceRNA network and PPI network. Then, we obtained the key genes for follow-up analysis.

### Functional enrichment (DEGs) and differential expression analysis (key genes)

Gene ontology (GO) functional annotation and Kyoto Encyclopedia of Genes and Genomes (KEGG) analysis were done for analyzing DEGs by using R language software package (Bioconductor and pathview) and DAVID online tool (version 6.8). P<0.05 indicated that the pathways or GO biological process terms were significantly enriched.

The present work adopted GraphPad Prism 8 (version 8.0.1) to visualize key DEGs in different datasets.

## Results

### Differential expression analysis

**Fig. 2:**
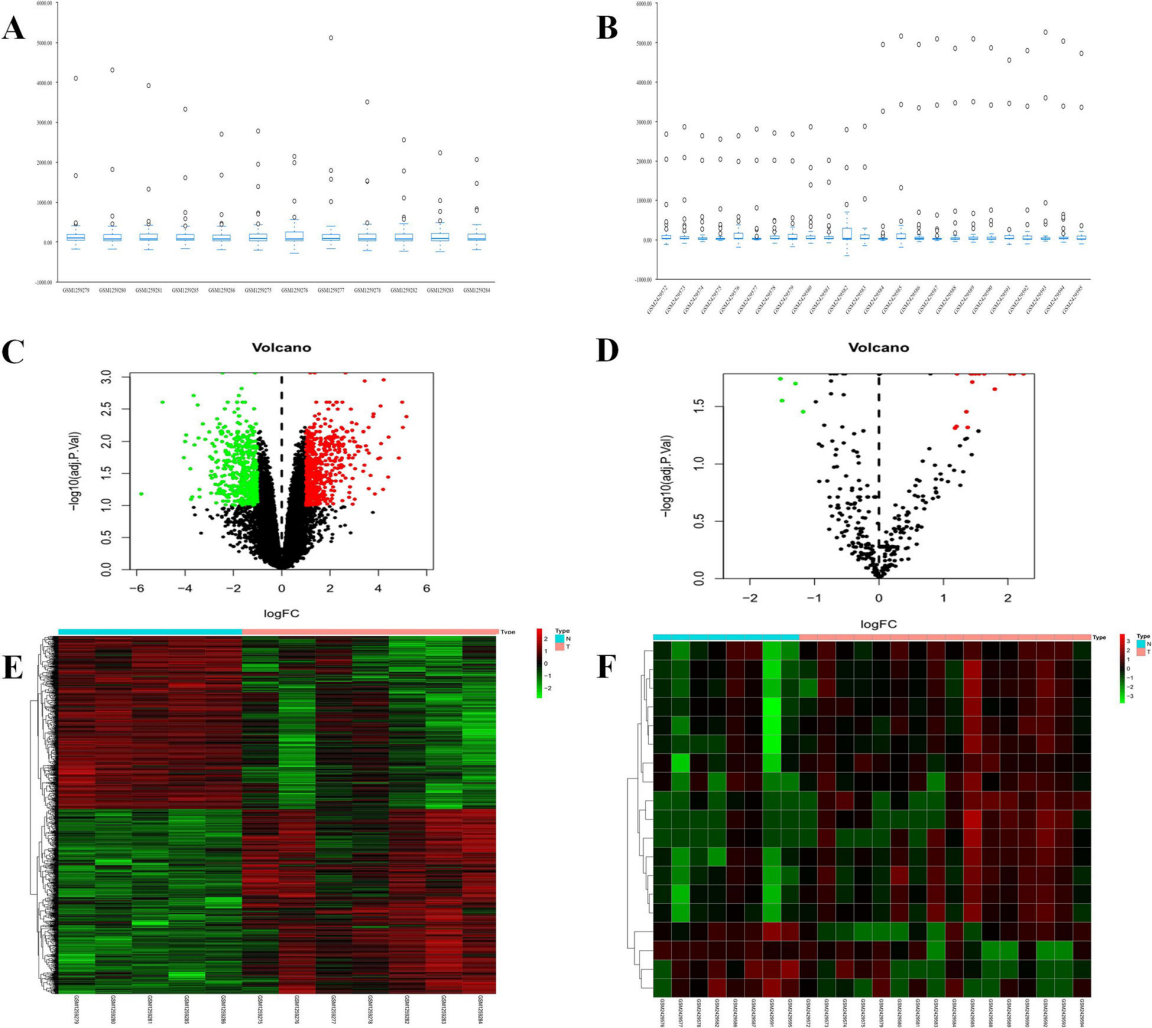
Differentially expressed analysis of LncRNAs, miRNAs and mRNAs. (**A**) Box plot of GSE52093, lncRNAs and mRNAs;(**B**) Box plot of GSE92427, miRNAs;(**C**) Volcano map of GSE52093, lncRNAs and mRNAs;(**D**) Volcano map of GSE92427, miRNAs;(**E**) heatmap analysis of GSE52093, lncRNAs and mRNAs;(**F**) heatmap analysis of GSE52093, miRNAs. Differentially expressed LncRNA and mRNA molecules were screened under the cut-off criteria log|FoldChange| > 1 and adjust*p* < 0.1, Differentially expressed miRNA molecules were screened under the cut-off criteria log|FoldChange| > 1 and adjust*p* < 0.05

In this study, we imported the corrected expression matrix data into the SPSSAU online software, and the resulting box plot was shown in **(**Figure 2A and B**)**. According to the cutoff criteria of GSE52093 (log|FoldChange|>1, adjust*p*<0.1) and the cutoff criteria of GSE92427 (log|FoldChange|>1, adjust*p*<0.05), volcano map and heat map for differential expressed lncRNAs, miRNAs and mRNAs were obtained **(**Figure 2C, D, E and F**)**. The GSE52093 dataset included lncRNA and mRNA data, in which three DELs (two with up-regulation and one with down-regulation) and 1046 DEmRNAs (540 with up-regulation and 506 wtih down-regulation) were discovered through the comparison of gene expression data between AD and control samples. Altogether 19 DEmiRs of GSE92427 dataset (15 upregulated and four downregulated) were identified from plasma samples including acute aortic dissection and healthy subjects. The most significant differentially expressed genes were shown in **(**Table 1**)**.

**Table 1:** The most significant differentially expressed genes, DELs and DEmiRs between normal tissue and aortic dissection

GSE52093					GSE92427				
ID	Type	logFC	*P*-Value	adj. P.Val	ID	type	logFC	P-Value	adj. P.Val
UCA1	lncRNA	−2.3374	3.09E-05	0.0059	hsa-let-7i	miRNA	2.2338	1.05E-03	0.0164
C9orf106	lncRNA	1.2121	1.18E-03	0.0224	hsa-miR-15a	miRNA	2.0280	2.37E-03	0.0164
FAM87A	lncRNA	1.7219	8.73E-03	0.0598	hsa-miR-15b	miRNA	1.6240	4.39E-03	0.0164
PHLDA1	mRNA	2.6381	1.27E-07	0.0009	hsa-miR-16	miRNA	1.5464	5.69E-03	0.0164
TIMP1	mRNA	1.1795	2.91E-07	0.0009	hur_4	miRNA	2.0861	6.98E-03	0.0164
LHFPL2	mRNA	1.3613	3.47E-07	0.0009	hsa-miR-107	miRNA	1.4910	8.12E-03	0.0164
CFL2	mRNA	−1.1051	3.61E-07	0.0009	hsa-let-7b	miRNA	1.4478	8.13E-03	0.0164
RYR2	mRNA	−2.4497	3.94E-07	0.0009	hsa-miR-29c	miRNA	1.4164	8.92E-03	0.0164
GINS2	mRNA	4.2148	6.07E-07	0.0011	hsa-miR-451	miRNA	1.2030	1.25E-02	0.0164
TMEM158	mRNA	3.4326	7.39E-07	0.0012	hsa-miR-494	miRNA	−1.5361	1.41E-02	0.0182
C5orf24	mRNA	−1.6611	1.11E-06	0.0015	hsa-miR-20a	miRNA	1.4397	1.50E-02	0.0194
JAK2	mRNA	−1.7771	1.66E-06	0.0020	hsa-miR-149*	miRNA	−1.3025	1.55E-02	0.0200
REEP1	mRNA	−3.6477	1.78E-06	0.0020	hsa-let-7 g	miRNA	1.7889	1.74E-02	0.0224
CASZ1	mRNA	−1.6201	2.69E-06	0.0025	hsv1-miR-H16	miRNA	−1.5109	2.20E-02	0.0282
TUBB3	mRNA	4.9842	3.06E-06	0.0025	hsa-miR-25	miRNA	1.3479	2.75E-02	0.0352
THSD4	mRNA	−1.8517	3.50E-06	0.0025	hsv1-miR-H1	miRNA	−1.1841	2.75E-02	0.0352
TSPAN5	mRNA	1.4282	3.59E-06	0.0025	hsa-let-7c	miRNA	1.1941	3.71E-02	0.0473
ECT2	mRNA	2.2850	3.72E-06	0.0025	hsa-miR-101	miRNA	1.3675	3.79E-02	0.0483
ACTC1	mRNA	−4.9309	3.87E-06	0.0025	hsa-miR-17	miRNA	1.1716	3.84E-02	0.0489

### Forecast of miRNA-mRNA and lncRNA-miRNA pairs

**Fig. 3:**
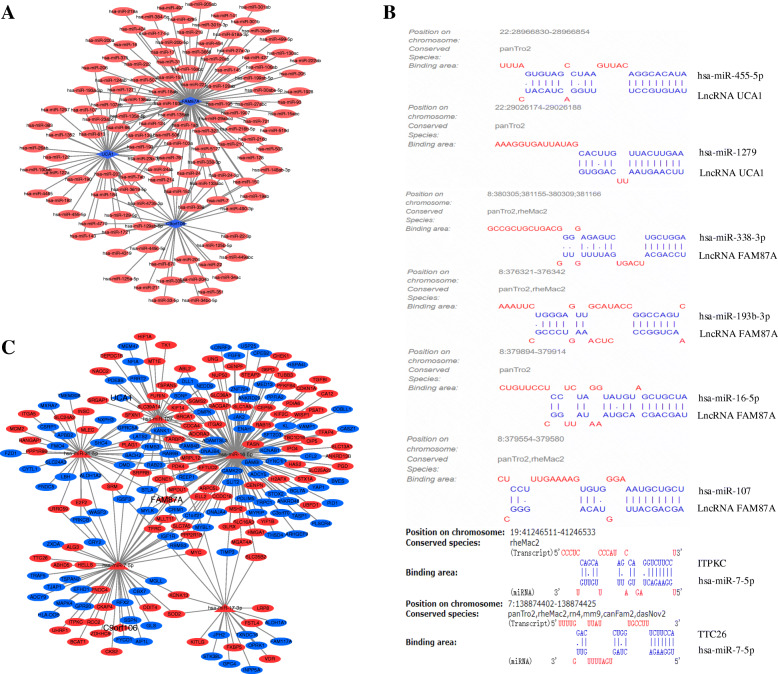
The analysis view of lncRNA, miRNA, mRNA target prediction and ceRNA network. (**A**) The blue quadrilateral represented lncRNA, the red oval represented miRNA that could interact with lncRNA;(**B**) lncRNA and predicted miRNA, miRNA and predicted mRNA binding site;(**C**) lncRNA-miRNA-mRNA ceRNA network, The quadrilateral: lncRNA, the triangle: miRNA, and the oval: mRNA. Red: up-regulation, blue: down-regulation

Interactive relationships between DELs and miRNAs were predicted with lncRNA mircode and R language script. We acquired the common miRNAs through the intersection of estimated DELs with DemiRs, while interactions of LncRNAs with miRNAs were predicted by LncBase Predicted v.2. LncRNA FAM87A can bind to hsa-miR-338-3p and hsa-miR-193b-3p. LncRNA UCA1 can bind to hsa-miR-1279 and hsa-miR-455-5p **(**Figure 3A**)**. Several DemiRs binding sites that can bind to lncRNA were presented in **(**Figure 3B**)**.

Compared with lncRNA prediction, the interaction relationships between DemiRs of binding DELs and DEGs were predicted with three databases form miRTarBase, TargetScan and miRDB. The screening conditions through the R language software package were set as follows. If the intersection was greater than 2, the DEGs interacting with DemiRs of binding DELs were exported. For instance, hsa-miR-107 can bind to SNGG (Synuclein Gamma) and CC2D1B (Coiled-Coil and C2 Domain Containing 1B). In addition, hsa-miR-16-5p can bind to LARP1 (La Ribonucleoprotein 1, Translational Regulator) and KCTD8 (Potassium Channel Tetramerization Domain Containing 8) **(**Table 2**)**. MicroT-CDS was employed for predicting the associations of miRNAs with mRNAs. Several DEGs binding sites that can bind to DemiRs were illuminated in **(**Figure 3B**)**.

**Table 2:** DEmiRs that have at least two predicted target mRNA modified

miRNA	Gene	miRDB	miRTarBase	TargetScan	Sum
hsa-miR-16-5p	LARP1	0	1	1	2
hsa-miR-107	SNCG	1	1	1	3
hsa-miR-107	CC2D1B	1	0	1	2
hsa-miR-16-5p	DNAJC15	0	1	1	2
hsa-miR-16-5p	ITGBL1	1	0	1	2
hsa-miR-17-3p	ARID4B	0	1	1	2
hsa-miR-16-5p	TUBGCP2	0	1	1	2
hsa-miR-17-3p	FAM19A1	1	0	1	2
hsa-miR-7-5p	MYLK	0	1	1	2
hsa-miR-107	HERC2	1	0	1	2
hsa-miR-16-5p	KCTD8	1	0	1	2
hsa-miR-7-5p	RELA	1	1	1	3
hsa-miR-107	RAB11FIP2	1	0	1	2
hsa-miR-107	SCAMP5	1	0	1	2
hsa-miR-17-3p	MTUS2	1	0	1	2
hsa-miR-16-5p	PRKAR2A	1	1	1	3
hsa-miR-7-5p	ARL15	0	1	1	2
hsa-miR-107	GLUD1	1	0	1	2
hsa-miR-17-3p	RNF11	0	1	1	2
hsa-miR-107	AMMECR1	1	0	1	2
hsa-miR-107	ENPP4	1	0	1	2
hsa-miR-7-5p	SEMA6D	0	1	1	2
hsa-miR-16-5p	ZC3H11A	0	1	1	2
hsa-miR-16-5p	AREL1	1	0	1	2
hsa-miR-16-5p	DOLPP1	1	0	1	2
hsa-miR-31-5p	AKNA	0	1	1	2
hsa-miR-16-5p	CLEC2D	0	1	1	2
hsa-miR-16-5p	CDC5L	0	1	1	2
hsa-miR-7-5p	SLC25A39	0	1	1	2

miRDB, miRTarBase, TargetScan: Three databases for predicting miRNA, the number represents the frequency of occurrence; Sum: The sum of the frequency of occurrence of the three databases

### lncRNA-miRNA-mRNA ceRNA network construction

Cytoscape (version 3.7.2) was employed to visualize the interaction between three DELs (two with up-regulation and one with down-regulation), five DemiRs (five with up-regulation) and 211 DEGs (103 with up-regulation and 108 with down-regulation). Therefore, the lncRNA-miRNA-mRNA ceRNA network was built, where LncRNA C9orf106 can be linked by one DEmiR and 51 DEGs, LncRNA UCA1 can be linked by one DEmiR and 42 DEGs and LncRNA FAM87A can be linked by three DEmiR and 185 DEGs **(**Figure 3C**)**. It includes LncRNA C9orf106-hsa-miR-7-5p-MYLK (Myosin Light Chain Kinase)/TJAP1 (Tight Junction Associated Protein 1); LncRNA FAM87A- hsa-miR-16-5p-MYC (MYC Proto-Oncogene, BHLH Transcription Factor)/H2AFX (H2A.X Variant Histone), LncRNA UCA1- hsa-miR-107-CDCA4(Cell Division Cycle Associated 4)/PDK4(Pyruvate Dehydrogenase Kinase 4) and LncRNA FAM87A-hsa-miR-17-3p-LRP8 (LDL Receptor Related Protein 8)/TFRC (Transferrin Receptor).

### PPI network analysis and key gene prediction

**Fig. 4:**
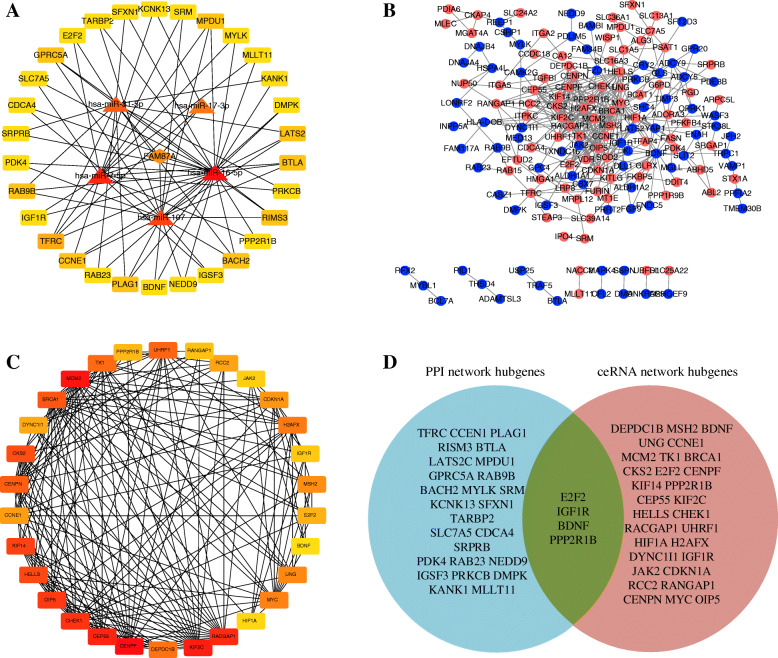
PPI network and key genes analysis. (**A**) Relationship network diagram of hub genes from ceRNA network;(**B**) PPI network. Red: up-regulated, Blue: down-regulated;(**C**) Relationship network diagram of hub genes from PPI network;(D) Venn diagram of key genes

The top 30 DEGs of ceRNA network acquired based on degree, closeness centrality and betweenness centrality algorithms using cytoHubba were visualized in **(**Figure 4A**)**. When uploading the DEGs identified in ceRNA network to STRING website, 395 interaction relationships were found to be present in 211 DEGs incorporated in constructing a PPI network. In the as-constructed network, E2F2(E2F Transcription Factor 2) could interact with CCNE1(Cyclin E1). IGF1R (Insulin Like Growth Factor 1 Receptor) could interact with BNDF (Brain Derived Neurotrophic Factor). BDNF could interact with IGF1R and PPP1R9B (Protein Phosphatase 1 Regulatory Subunit 9B) **(**Figure 4B**)**. The top 30 DEGs of PPI network obtained by cytoHubba were visualized in **(**Figure 4C**)**. There were four key gens (E2F2, IGF1R, BDNF and PPP1R9B) which were determined by Venn diagram of ceRNA network and PPI network **(**Figure 4D**)**.

### Functional enrichment and differential expression analysis

**Fig. 5:**
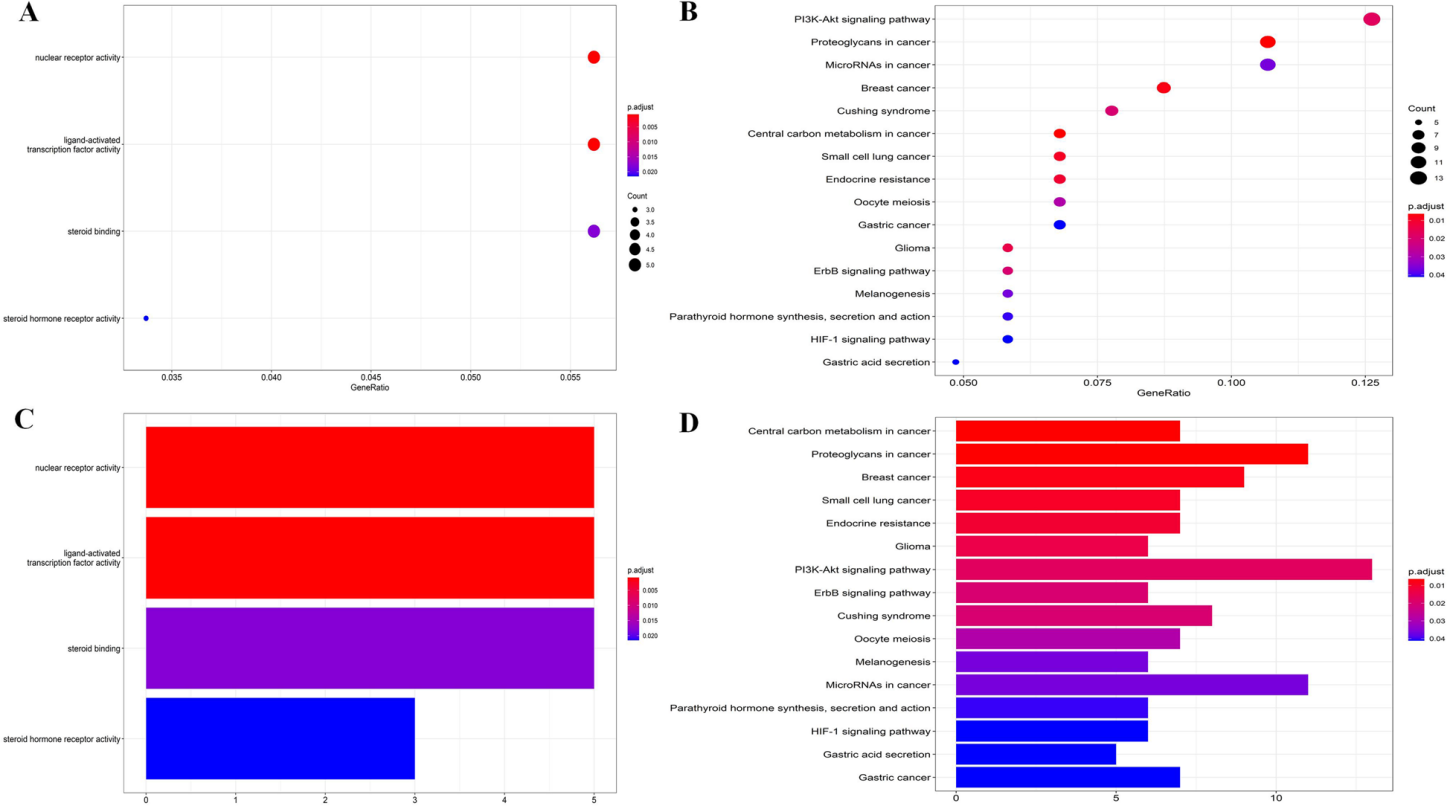
GO/KEGG analysis of DEGs. (**A**) Bubble chart of GO. Each bubble represented a function;(**B**) Bubble chart of KEGG. Each bubble represents a pathway;(**C**) Histogram of GO;(**D**) Histogram of KEGG

In the present study, the DEGs used to analyze GO/KEGG were imported into DAVID website and R language script. The results of DAVID showed the functions and pathways involved in DEGs: GO (such as plasma membrane and protein binding) and KEGG (like pathways in cancer and PI3K-Akt signaling pathway) **(**Table 3**)**. The results of R language software showed the functions and pathways involved in DEGs: GO (such as nuclear receptor activity and steroid binding) and KEGG (such as proteoglycans in cancer and PI3K-Akt signaling pathway) **(**Figure 5**)**.

**Table 3:** GO/KEGG analysis of DEGs

Term of GO/KEGG analysis	Count	P-Value	Genes
GO:0045893 ~ positive regulation of transcription, DNA-templated	16	0.001254	FZD1, HMGA1, BRCA1, MRPL12, HIF1A, MLLT11, ZXDA, LBH, TFAP4, MED13, CCNE1, BAMBI, MYC, CKS2, TASP1, MYBL1
GO:0008283 ~ cell proliferation	13	0.001433	YAP1, UHRF1, ITGA2, FURIN, CENPF, KITLG, MYC, DDIT4, CKS2, KIF2C, TGFBI, GPC4, BCAT1
GO:0007067 ~ mitotic nuclear division	10	0.002886	HELLS, CENPF, LATS2, TUBB3, RCC2, NEDD9, OIP5, KIF2C, CENPN, CEP55
GO:0051301 ~ cell division	11	0.009168	HELLS, CENPF, LATS2, CCNE1, CDCA4, RCC2, KIF14, CKS2, NEDD9, OIP5, KIF2C
GO:0098609 ~ cell-cell adhesion	8	0.042913	TMEM47, LRRC59, GPRC5A, FASN, COBLL1, UBFD1, RANGAP1, PDLIM5
GO:0005886 ~ plasma membrane	69	0.000237	ANKRD13B, TFRC, KIF14, GPR20, BRCA1, IGF1R, PPP1R9B, TMEM47, JPH2, ADORA3, BVES, SLC16A3, SLC36A1, SLC13A1, PRKCB, TRPC1, ITGA2, KCNK13, KCNAB1, PDIA6, SLC39A14, CKAP4, ENAH, FNDC5, SLC7A5, FNDC4, GPRC5A, ADCY9, PLSCR4, ITGA5, STX1A, MGLL, FAM84B, SLC24A2, SHC4, KANK1, SLC24A3, IGSF3, FURIN, SGMS2, SLC1A5, DLL1, LRP8, ADCY5, INPP5A, RAB23, DNAJB4, BTLA, TSPAN5, DMD, SLIT2, GPC4, CAMK2G, FZD1, CA12, KL, PRRT2, TMEM30B, DMPK, RCC2, CRIM1, OPRK1, TJAP1, KITLG, BAMBI, RAB15, FASN, TGFBI, RAB9B
GO:0005829 ~ cytosol	58	0.000335	CDKN1A, KIF14, MYLK, GLS, MYC, CHEK1, TK1, JAK2, PDE8B, PPFIA2, G6PD, PRKCB, KCNAB1, RANGAP1, PGD, ENAH, ITPKC, SLC7A5, ARHGEF9, DEPDC1B, LATS2, CCNE1, ALDH1A2, DDIT4, TRAF5, ALDH1A1, VAMP1, KIF2C, BCAT1, STX1A, MGLL, YAP1, PFKFB4, HSPA4L, GLRX, ABHD5, HIF1A, SRM, RACGAP1, DNAJB4, ABL2, DMD, PDLIM5, SRGAP1, CAMK2G, MAPK4, DYNC1I1, TBC1D16, DMPK, RCC2, HMGA1, TARBP2, CENPF, PSAT1, DNAJA4, FASN, CENPN, RAB9B
GO:0045202 ~ synapse	9	0.000975	ENAH, PRRT2, OPRK1, APBB2, DMD, MYRIP, ITGA5, CPEB2, PPFIA2
GO:0016020 ~ membrane	41	0.001156	YAP1, SLC35B2, LRRC59, SFT2D3, TFRC, KIF14, FURIN, SLC1A5, LRP8, IPO4, IGF1R, EFTUD2, FYCO1, INPP5A, MED13, MLEC, STK38L, APBB2, SLIT2, JAK2, PDLIM5, SLC16A3, CAMK2G, CEP55, MPDU1, G6PD, PRRT2, RCC2, CKAP4, REEP1, SLC7A5, KITLG, MSH2, DNAJA4, FASN, MGAT4A, SRPRB, SFXN1, KIF2C, MGLL, FKBP5
GO:0030027 ~ lamellipodium	8	0.002131	ENAH, KITLG, NEDD9, APBB2, SLC39A14, WASF3, MYLK, PPP1R9B
GO:0070062 ~ extracellular exosome	45	0.010861	TFRC, ARPC5L, FURIN, GLRX, SLC1A5, THSD4, TXNDC16, MYLK, INPP5A, RACGAP1, FGF9, PPP2R1B, CSRP1, TUBB3, RAB23, DNAJB4, CFL2, COBLL1, TIMP3, EFHD1, SLIT2, GPC4, WASF3, PPFIA2, MPDU1, KL, G6PD, PRKCB, H2AFX, CRIM1, PGD, SOD2, PDIA6, CKAP4, AIF1L, SLC7A5, GPRC5A, RAB15, PSAT1, FASN, MGAT4A, PLSCR4, ALDH1A1, TGFBI, FKBP5
GO:0005737 ~ cytoplasm	73	0.020204	ANKRD13B, STEAP3, PID1, ARPC5L, BRCA1, WISP1, BACH2, IPO4, MYLK, PPP1R9B, EFTUD2, LBH, FGF9, TUBB3, OIP5, JAK2, PPFIA2, G6PD, PRKCB, RFX2, KCNAB1, RANGAP1, ENAH, SLC7A5, ARHGEF9, LATS2, NUP50, ALDH1A2, CRY2, DDIT4, TRAF5, ALDH1A1, MT1E, RBMS3, FAM84B, MCM2, YAP1, KANK1, CASZ1, HSPA4L, NEDD9, ABHD5, HIF1A, FAM117A, RACGAP1, RAB23, DNAJB4, HAS2, STK38L, APBB2, SLIT2, PDLIM5, SRGAP1, WASF3, MAPK4, DYNC1I1, USP25, CBX7, BDNF, ELL2, TARBP2, AIF1L, REEP1, MLLT11, KITLG, CENPF, ZDHHC9, PSAT1, BAMBI, RAB15, FASN, SRPRB, CPEB2
GO:0005887 ~ integral component of plasma membrane	25	0.025690	SLC24A2, STEAP3, SLC24A3, TFRC, GPR20, SGMS2, SLC1A5, DLL1, IGF1R, HAS2, TSPAN5, TSPAN2, GPC4, SLC16A3, SLC13A1, KL, TRPC1, KCNK13, OPRK1, SLC39A14, SLC7A5, GPRC5A, ADCY9, SSPN, VAMP1
GO:0019899 ~ enzyme binding	11	0.004383	MSH2, PLSCR4, H2AFX, HMGA1, MLEC, TSPAN5, BRCA1, HIF1A, TARBP2, MCM2, UNG
GO:0005515 ~ protein binding	112	0.037391	STEAP3, PID1, TFRC, IPO4, TXNDC16, MYLK, IGF1R, GLS, EFTUD2, ZXDA, PPP2R1B, TUBB3, MYC, CFL2, CHEK1, OIP5, BVES, SLC16A3, PPFIA2, G6PD, PRKCB, RFX2, PDIA6, MSH2, CCNE1, STX1A, FKBP5, SHC4, CDCA4, SLC1A5, MRPL12, HIF1A, INPP5A, RACGAP1, NACC2, ABL2, STK38L, TSPAN2, SRGAP1, MPDU1, TBC1D16, FZD1, CBX7, DMPK, VDR, RCC2, HMGA1, TJAP1, REEP1, FASN, TGFBI, CDKN1A, KIF14, BRCA1, PPP1R9B, FYCO1, JPH2, BCL7A, ADAMTSL3, TIMP3, TK1, JAK2, CEP55, HELLS, TRPC1, ITGA2, H2AFX, MYRIP, RANGAP1, ENAH, GPRC5A, LATS2, NUP50, PLSCR4, TRAF5, CRY2, CKS2, VAMP1, KIF2C, ITGA5, MCM2, FAM84B, YAP1, KANK1, UHRF1, NEDD9, FURIN, DLL1, LRP8, SRM, UNG, RAB23, DNAJB4, E2F2, APBB2, DMD, SLIT2, PDLIM5, CAMK2G, MAPK4, DYNC1I1, USP25, TMEM30B, OPRK1, C1ORF21, TARBP2, CENPF, KITLG, TFAP4, DNAJA4, RAB15, RAB9B
hsa05230:Central carbon metabolism in cancer	7	0.000246	SLC7A5, G6PD, MYC, SLC1A5, HIF1A, SLC16A3, GLS
hsa05200:Pathways in cancer	16	0.000319	FZD1, CDKN1A, PRKCB, ITGA2, HIF1A, ADCY5, IGF1R, KITLG, ADCY9, FGF9, MSH2, CCNE1, MYC, TRAF5, CKS2, E2F2
hsa05205:Proteoglycans in cancer	11	0.000441	FZD1, CDKN1A, TFAP4, PRKCB, MYC, ITGA2, TIMP3, ITGA5, CAMK2G, HIF1A, IGF1R
hsa05214:Glioma	6	0.002033	SHC4, CDKN1A, PRKCB, E2F2, CAMK2G, IGF1R
hsa04114:Oocyte meiosis	7	0.004406	ADCY9, PPP2R1B, CCNE1, CPEB2, CAMK2G, IGF1R, ADCY5
hsa05206:MicroRNAs in cancer	11	0.006299	SHC4, CDKN1A, CCNE1, PRKCB, MYC, DDIT4, TIMP3, E2F2, BRCA1, ITGA5, GLS
hsa05222:Small cell lung cancer	6	0.006490	CCNE1, MYC, ITGA2, TRAF5, CKS2, E2F2
hsa04012:ErbB signaling pathway	6	0.007153	SHC4, CDKN1A, PRKCB, MYC, ABL2, CAMK2G
hsa04151:PI3K-Akt signaling pathway	12	0.008276	CDKN1A, KITLG, FGF9, PPP2R1B, CCNE1, MYC, ITGA2, DDIT4, BRCA1, ITGA5, JAK2, IGF1R
hsa04066:HIF-1 signaling pathway	6	0.010730	CDKN1A, TFRC, PRKCB, CAMK2G, HIF1A, IGF1R
hsa04916:Melanogenesis	6	0.012655	FZD1, KITLG, ADCY9, PRKCB, CAMK2G, ADCY5
hsa04971:Gastric acid secretion	5	0.018626	ADCY9, PRKCB, CAMK2G, MYLK, ADCY5
hsa04110:Cell cycle	6	0.029203	CDKN1A, CCNE1, MYC, CHEK1, E2F2, MCM2
hsa05414:Dilated cardiomyopathy	5	0.029430	ADCY9, ITGA2, DMD, ITGA5, ADCY5
hsa04911:Insulin secretion	5	0.030563	ADCY9, PRKCB, CAMK2G, STX1A, ADCY5
hsa04310:Wnt signaling pathway	6	0.043260	FZD1, PRKCB, BAMBI, MYC, GPC4, CAMK2G

**Fig. 6:**
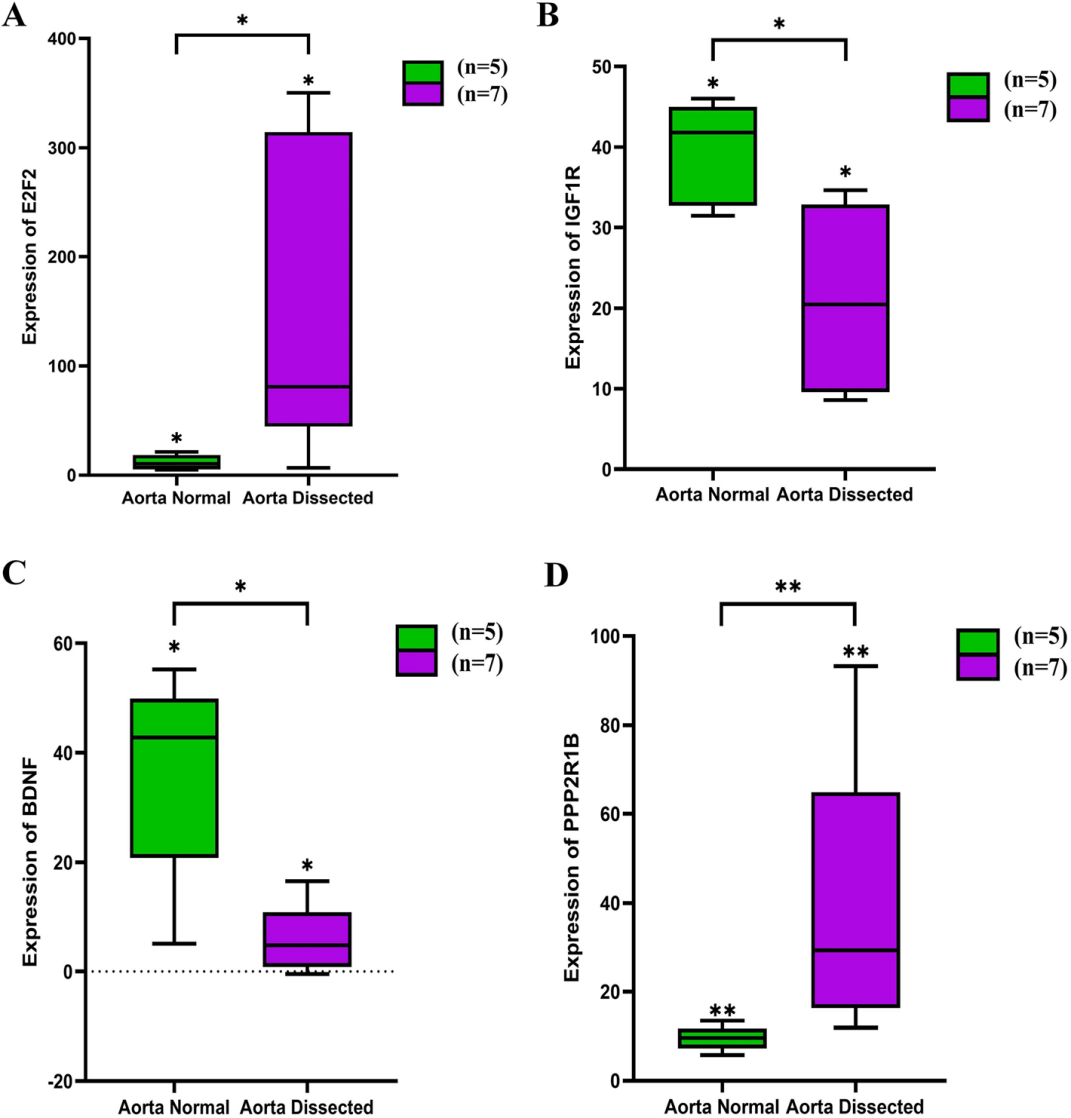
Difference Analysis Box Plot of key genes. (**A**) Expression of E2F2 between normal aorta and aortic dissection; (**B**) Expression of IGF1R between normal aorta and aortic dissection; (**C**) Expression of BDNF between normal aorta and aortic dissection; (**D**) Expression of PPP2R1B between normal aorta and aortic dissection. Green represented normal aortic samples, and purple represented aortic dissection samples

To accurately explore the differential expression of key genes in the three datasets, four key genes were visualized in **(**Figure 6**).** In the GSE52093 datasets, E2F2 and PPP1R9B were up-regulated while IGF1R and BDNF were down-regulated, further revealing the underlying mechanisms for key genes.

## Discussion

In normal circumstances, the 3 layers of aortic wall (e.g. intima, media, and adventitia) should maintain the complete structures and physiological functions for maintaining the aortic wall stability and managing the great influence resulting from blood flow [17]. The AD pathogenic mechanisms are vascular inflammation, matrix metalloproteinases (MMPs) activity and change in vascular smooth muscle cells (VSMCs) phenotype [18]. Endothelial cell (EC) injury promotes the occurrence of vascular inflammation modulated via immune response. Afterwards, it will activate MMP, the extracellular matrix (ECM) degrading enzyme [19, 20]. Meanwhile, AD is also associated with broad alterations of VSMC phenotype and vascular wall at molecular level [21, 22].

LncRNAs, with over 200 nucleotides in length, constitute the transcript family without protein coding functions [23]. They are suggested to play roles of ceRNAs for miRNAs; in other words, lncRNAs may play roles of miRNA “decoys” for regulating gene levels [12, 24]. As suggetsed by the theory, lncRNAs act as natural sponge for the competition adsorption of certain miRNAs and reduction of miRNA binding to corresponding target genes, thus resulting in alterations of miRNA target gene expression [25]. However, it remains unclear about whether the abnormal lncRNAs play roles of ceRNA for certain miRNAs and have certain influence on arterial wall by the indirect regulation of target mRNA expression in the process of AD occurrence.

Recently, lncRNAs have been discovered to be related to CVD genesis and progression. Recent study revealed a pathogenic H19 induce aneurysm by inflammatory pathway related to the formation of AAA, and this has offered a novel treatment for AAA [26]. Meanwhile, Kumar and colleagues investigated the effects of ncRNAs together with the target genes from the perspective of their functions within AAA. They also discussed those animal models adopted to mechanically understand AAA and the possible effects of miRNAs and lncRNAs as diagnostic biomarker and therapeutic targets [27]. Several lncRNAs are suggested to have important functions in aorta-related disease pathogenesis, such as AD [28]. Few lncRNAs show obvious spatiotemporal expression and specificity in the process of tissue growth and differentiation; As a result, they were the favorable diagnostic biomarkers for AD.

E2F belongs to the transcriptional factor family, which functions in controlling G(1)/S transition. In addition, certain E2F components have been recently suggested to regulate functions in addition to cell cycle, like apoptosis induction [29]. Fujita et al. found that E2F5 was induced by fetal bovine serum in VSMC, and E2F5 inducibility has unique function in VSMC, which is related to the feedback modulation for some cell activities in cell proliferation [30]. Nevertheless, Angiotensin II (ANG II) increased E2F3 protein expression, rather than E2F-5 and E2F-1, but it did not increase the mRNA expression. The above alterations are related to the hyperplastic or hypertrophic responses to diverse stimuli or growth factors of VSMCs. Several studies suggested that modulation of E2F had certain effect on treating hypertension, atherosclerosis, restenosis following vascular damage and bypass graft failure [31, 32]. In precent study, E2F2 expression was possibly modulated via lncRNA FAM87A-hsa-miR-31-5p/hsa-miR-7-5p in human aortic dissection tissue. Recent study investigated the implication overexpression miR-7-5p, finding that miR-7-5p agomir transfection markedly suppressed mineralization of pulmonary artery smooth muscle cell matrix under hypoxic conditions [33].

It is still unclear about the AD pathogenesis, but more and more studies have suggested the important functions of ncRNAs within AD. In this study, the underlying molecular mechanisms of AD have been investigated based on the lncRNA-miRNA-mRNA ceRNA regulatory network. lncRNAs, mRNAs and miRNAs expression data are obtained from human tissues of dissection and normal groups. Wang et al. discovered the abnormally expressed miRNAs and lncRNAs within AD samples and identified the role of lncRNA OIP5-AS1 in exacerbating injuries to all the three layer of aortic wall in AD genesis and development by increasing TUB expression through the sponge of miR-143-3p [34]. As discovered by another study, lncRNA PVT1 level increased, yet miR-27b-3p level decreased within AD tissue. They reduced the lncRNA PVT1 level to suppress the migration, phenotype switch and viability of human aortic smooth muscle cells treated with growth factor-BB (PDGF-BB) through targeting miR-27b-3p [35]. For understanding the pathophysiological and biological mechanisms related to AD genesis and development, circRNAs, miRNAs, and lncRNAs with abnormal expression should be discovered and verified in relevant human AD samples or relevant animal models. Extraction of total RNA from critical types of cells, like immune cells, ECs, and SMCs, can shed more lights on alterations specific to cell type in the process of AD progression. When the differential expression is verified, it is necessary to test those mechanisms of ncRNAs in regulating AD progression in vitro and in vivo at molecular level.

Certain limitations should be noted in the present work. At first, although it has been shown that E2F2, IGF1R, and BDNF can cause arterial-related diseases, there is no research to prove the relationship between aortic dissection and these target gene. Secondly, the way of action has been predicted based on the measured RNA network, however, which has not been confirmed (dual luciferase reporter gene analysis, gene overexpression or gene knockout). Although several related genes have been screened out in the present study for the first time, further in vitro clinical research and in vivo experiments should be carried out to confirm its expression and functional mechanism in terms of AD.

Currently, little research is conducted to explore the lncRNA mechanism in AD. The present work has its certain strengths. For instance, it is the first study to construct the lncRNA-miRNA-mRNA network based on GEO database. Nonetheless, our findings were just obtained from bioinformatics analysis. Therefore, it is of necessity to conduct a thorough study for verifying the potential effects of those 7 axes within AD. In summary, we confirmed that the ceRNA networks, including the regulated networks, lncRNA FAM87A-hsa-miR-31-5p/hsa-miR-7-5p-E2F2, lncRNA C9orf106-hsa-miR-7-5p-IGF1R, and lncRNA UCA1-hsa-miR-107-BDNF, might be associated with the pathogenesis of and development of AD. Moreover, our study shed novel lights on the AD pathogenic mechanism. Nevertheless, ceRNA networks and their associations with AD should be validated.

## Conclusions

In summary, the ceRNA interaction axis we identified is a potentially critical target for treating AD. According to the difference significance, PPI interaction correlation and regulation relationship, E2F2 axis (LncFAM87A-hsa-miR-7-5p/hsa-miR-31-5p-E2F2) may play a key role in the treatment of AD. Our results shed more lights on the possible pathogenic mechanism in AD using a lncRNA-associated ceRNA network.

## Data Availability

The datasets generated and analyzed during the current study are available in the Gene Expression Omnibus (GEO, https://www.ncbi.nlm.nih.gov/geo/) database (Accession Number: GSE52093, GSE92427).

## Abbreviations

AD: aortic dissection
LncRNA: Long non-coding RNA
GO: Gene ontology
hsa: *Homo sapiens*
KEGG: Kyoto encyclopedia of genes and genomes
mRNA: Messenger RNA
